# Dendrobium officinale polysaccharides ameliorate radiation-induced premature ovarian insufficiency through coordinated modulation involving the gut microbiota-PI3K/AKT-glycolysis axis

**DOI:** 10.3389/fphar.2026.1891795

**Published:** 2026-10-14

**Authors:** Bixiao Wei, Yanyun Huang, Xiaofang Huang, Jian Wang, Sheng Dou, Fei Yu, Liuyi Huang, Mingyou Dong, Junli Wang

**Affiliations:** 1 Clinical Laboratory, Affiliated Souhwest Hospital of Youjiang Medical University for Nationalies, Baise, Guangxi, China; 2 Guangxi Key Laboratory of Artificial Intelligence for Genetic Diseases of Long-dwelling Nationalities, Youjiang Medical University for Nationalities, Baise, Guangxi, China; 3 Reproductive Medicine Center, The People’s Hospital of Baise, Baise, Guangxi, China; 4 Department of Radiology, The People’s Hospital of Baise, Baise, Guangxi, China; 5 Key Laboratory of Precision Molecular Laboratory Medicine, Baise City, Affiliated Southwest Hospital of Youjiang Medical University, Baise, Guangxi, China; 6 Clinical Laboratory, The Afffliated Hospital of Youjiang Medical University for Nationalities, Baise, Guangxi, China

**Keywords:** dendrobium officinale polysaccharide, glycolysis, gut microbiota, PI3K/Akt pathway, premature ovarian insufficiency, radiotherap, y

## Abstract

Radiotherapy-induced premature ovarian insufficiency (POI) remains a clinical challenge with limited preventative strategies. Dendrobium officinale polysaccharides (DOP) exhibit beneficial bioactivities, but their efficacy and mechanism against radiation-induced POI are unknown. In this study, a mouse model of POI was established by single-dose whole-body X-ray irradiation (5 Gy). Mice were pretreated with a partially purified DOP preparation (500 mg/kg/day) for 7 days prior to and 14 days post-irradiation. Ovarian function was assessed via estrous cyclicity, hormone assays, histomorphology, and follicle counting. Mechanisms were investigated through analysis of apoptosis (TUNEL, IHC, flow cytometry), PI3K/AKT signaling and glycolysis-related marker expression (qPCR, IF, Western blot), and gut microbiota (16S rRNA sequencing), with *in vitro* validation using irradiated human granulosa cells (KGN). DOP pretreatment significantly attenuated POI phenotypes, restoring estrous cyclicity, improving serum sex hormone profiles (increased E2 and AMH; decreased FSH and LH), and preserving ovarian/uterine morphology and follicle pool. Mechanistically, DOP inhibited ovarian granulosa cell apoptosis *in vivo* and *in vitro*, concomitant with reduced ROS levels. Immunofluorescence and qPCR analyses revealed that DOP modulated the PI3K/AKT pathway in growing follicles while suppressing its overactivation in primordial follicles, as evidenced by modulated expression and localization of FOXO3a and rps6 and increased phosphorylation of AKT, mTOR, FOXO3a, and rps6. Concurrently, DOP upregulated the expression of glycolysis-related markers including GLUT4, HK1, PKM2, and LDHA at both mRNA and protein levels. Furthermore, DOP restored gut microbiota diversity and composition altered by irradiation, increasing the abundance of beneficial taxa such as Muribaculum. Collectively, these findings suggest that DOP exerts a protective effect against radiation-induced POI through a multi-targeted mechanism involving anti-apoptosis, modulation of PI3K/AKT signaling and glycolysis-related marker expression in growing follicles, inhibition of primordial follicle overactivation, and remediation of gut dysbiosis. These findings position DOP as a promising natural candidate for improving ovarian injury-related parameters, warranting further investigation.

## Introduction

Premature ovarian insufficiency (POI), defined as the loss of ovarian function before age 40, affects approximately 1%–2% of women worldwide and represents a leading cause of female infertility (Webber et al., 2016). Clinically, POI is characterized by secondary amenorrhea, elevated serum follicle-stimulating hormone (FSH > 25 IU/L), and estrogen deficiency, reflecting a severe depletion of the ovarian follicle reserve (Nash and Davies, 2024). Among its heterogeneous etiologies, radiotherapy-induced ovarian damage is particularly concerning, as pelvic irradiation is required for various malignancies. Approximately 30% of female cancer survivors who receive pelvic radiation develop POI, with the risk increasing with higher doses and younger age (Kim et al., 2021; Wo and Viswanathan, 2009; Meirow et al., 2010).

Ionizing radiation exerts its deleterious effects primarily through the generation of reactive oxygen species (ROS), which induce DNA damage, mitochondrial dysfunction, and activation of apoptotic cascades (Kim et al., 2014; Jiao et al., 2022). Radiation-induced ovarian damage occurs through the depletion of ovarian follicles and injury to oocytes via oxidative stress and direct DNA double-strand breaks, accelerating reproductive aging and leading to conditions such as POI (Lee et al., 2025; Cai, 2025; Ramirez and Viel, 2024). The primordial follicle pool, which represents the finite ovarian reserve, is exceptionally vulnerable to radiation because dormant oocytes lack robust antioxidant defenses (Oktem and Oktay, 2007). Moreover, radiation disrupts the ovarian stromal microenvironment and triggers chronic inflammation, accelerating follicular atresia (Du et al., 2022). Despite advances in assisted reproductive technologies, effective pharmacological agents to prevent or mitigate radiation-induced ovarian damage are still lacking, highlighting an urgent clinical need (Sellami et al., 2025). Recent transcriptomic and machine-learning analyses have identified candidate biomarkers for POI (Yu et al., 2023).

Natural polysaccharides from medicinal plants have attracted attention for their low toxicity and multi-targeted bioactivities (Zhao et al., 2020). Dendrobium officinale Kimura et Migo, a prized orchid in traditional Chinese medicine, is rich in bioactive polysaccharides with documented antioxidant, immunomodulatory, and gut microbiota-regulating properties (Tang et al., 2017; Chen et al., 2023). Notably, a DOP preparation has shown therapeutic effects in polycystic ovary syndrome models via modulating the gut-brain-ovary axis and inhibiting granulosa cell apoptosis (Feng et al., 2022). Additionally, a DOP preparation has been reported to protect against natural aging-induced premature ovarian failure by reducing p53 expression and increasing Bcl-2 expression in the ovaries of aging mice (Wu et al., 2018). However, whether DOP confers protection against radiation-induced POI remains completely unexplored.

Emerging evidence supports a “gut-ovary axis”, where gut dysbiosis influences systemic inflammation and endocrine signaling, potentially impacting ovarian physiology (Zhao et al., 2025; Qi et al., 2021). The PI3K/AKT signaling pathway is a central regulator of follicular activation, survival, and growth, but must be precisely controlled in a follicle stage-specific manner (Reddy et al., 2008; John et al., 2008; Castrillon et al., 2003; Adhikari et al., 2009). Additionally, glycolysis in granulosa cells is essential for supporting oocyte development (Sutton-McDowall et al., 2010). It remains unclear whether and how DOP orchestrates gut microbiota, PI3K/AKT signaling and follicular glycolysis-related processes to antagonize radiation-induced POI. Therefore, this study aimed to investigate the protective effects of a partially purified DOP preparation against X-ray-induced POI in a mouse model and elucidate the underlying mechanisms involving the gut microbiota-PI3K/AKT-glycolysis axis.

## Materials and methods

### Preparation and characterization of DOP

A partially purified polysaccharide-rich preparation was extracted and purified from dried stems of Dendrobium officinale according to a previously established protocol with slight modifications (Feng et al., 2022). Briefly, the process involved defatting with petroleum ether, hot-water extraction, ethanol precipitation, deproteinization by the Sevag method, dialysis, and purification via DEAE-52 cellulose column chromatography. The resulting preparation had a total polysaccharide content of 59.09%, as determined using the phenol-sulfuric acid method with glucose as a standard, indicating that the preparation also contained other unidentified components. The monosaccharide composition was analyzed by high-performance liquid chromatography (HPLC; Agilent 1260 series) after acid hydrolysis and 1-phenyl-3-methyl-5-pyrazolone (PMP) derivatization. The average molecular weight and distribution were determined by gel permeation chromatography (GPC) coupled with multi-angle laser light scattering (MALLS). As the present study used a single production batch, the claim of batch-to-batch consistency was not established; however, all quality control procedures including polysaccharide content determination, monosaccharide composition analysis, and molecular weight profiling were performed on this batch to ensure characterization accuracy.

### Animals and experimental design

All animal procedures were approved by the Animal Ethics Committee of Youjiang Medical College for Nationalities (Approval No.: 2022010701) and performed in strict compliance with the NIH Guide for the Care and Use of Laboratory Animals. Female ICR mice (8 weeks old, 28 ± 2 g) were purchased from Guangdong Vital River Laboratory Animal Technology Co., Ltd. and housed under specific pathogen-free (SPF) conditions with a controlled 12/12 h light/dark cycle, ambient temperature maintained at 22 °C ± 2 °C, and relative humidity at 50% ± 10%. Following 1 week of acclimatization, mice exhibiting regular estrous cycles were randomly assigned into three experimental groups (n = 10 per group) using a computer-generated randomization protocol: (1) Control group: received vehicle (saline) via oral gavage; (2) POI group (5 Gy): administered vehicle and subjected to a single dose of whole-body X-ray irradiation (5 Gy); (3) POI + DOP group (5 Gy + DOP): pretreated with the DOP preparation (500 mg/kg/day, dissolved in saline) via oral gavage for seven consecutive days prior to irradiation, with treatment continued for 14 days post-irradiation, and received the same irradiation protocol. The DOP preparation dosage of 500 mg/kg/day was selected based on previously published studies demonstrating the efficacy and safety of this dose in mouse models of ovarian dysfunction (Feng et al., 2022; Wu et al., 2018) and other disease models (Zhong et al., 2022). Whole-body irradiation was delivered using an RS2000 biological irradiator (Rad Source Technologies, United States) at a dose rate of 1.2 Gy/min with a source-to-skin distance of approximately 50 cm. Mice in the control group underwent sham irradiation. Body weight and general health status were monitored daily throughout the experimental period. At the end of the experiment, mice were euthanized by cervical dislocation under deep anesthesia, with death confirmed by respiratory arrest and loss of heartbeat.

### Estrous cycle monitoring

Vaginal cytology was performed daily between 9:00 and 10:00 a.m. for 14 consecutive days until sacrifice. Vaginal lavage fluid was collected, smeared onto glass slides, air-dried, fixed in methanol, and stained with Wright-Giemsa stain (Solarbio, China). Estrous cycle stages (proestrus, estrus, metestrus, diestrus) were determined based on the predominant cell types (nucleated epithelial cells, cornified squamous cells, leukocytes) under a light microscope (Nikon Eclipse E100) by two independent, blinded observers. For each mouse, the proportion of days spent in each stage over the 14-day monitoring period was calculated. These stage proportions were then compared among groups using the Kruskal-Wallis test followed by Dunn’s *post hoc* test, as the data did not meet the assumptions of normality. This approach treated each animal as an independent unit of analysis and summarized the repeated daily observations as a single proportion per stage per animal.

### Sample collection and hormone measurement

On day 14 post-irradiation, mice were anesthetized with an intraperitoneal injection of 10% chloral hydrate (0.2 mL/100 g body weight). Deep anesthesia was confirmed by the absence of response to toe pinch. Blood was collected via terminal retro-orbital blood collection under deep anesthesia, allowed to clot, and centrifuged at 2,500×g for 20 min at 4 °C to obtain serum. Serum levels of estradiol (E2, #LV30673), follicle-stimulating hormone (FSH, #LV30585), luteinizing hormone (LH, #LV30679), and anti-Müllerian hormone (AMH, #LV30586) were quantified using specific mouse ELISA kits (Aimeng Youning, Shanghai, China) according to the manufacturer’s instructions. Ovaries and uteri were dissected, weighed to calculate organ indices (organ weight/body weight × 100%), and processed for subsequent analyses.

### Histopathological analysis

Ovarian and uterine tissues were fixed in 4% paraformaldehyde overnight, embedded in paraffin, and sectioned at 5 μm thickness. Sections were deparaffinized, rehydrated, and stained with Hematoxylin and Eosin (H&E). Ovarian morphology was examined, and follicles at different stages (primordial, primary, secondary, antral, atretic) were classified and counted in every fifth serial section throughout the entire ovary by two blinded investigators. Only follicles containing a visible oocyte nucleus were counted to avoid double-counting. The total number of sections examined per ovary ranged from 25 to 35, and both ovaries from each animal were analyzed. Uterine sections were assessed for endometrial thickness and glandular morphology.

### Immunohistochemistry (IHC)

Paraffin-embedded ovarian sections were subjected to antigen retrieval in citrate buffer (pH 6.0). Endogenous peroxidase activity was quenched with 3% H_2_O_2_. After blocking with 5% bovine serum albumin (BSA), sections were incubated overnight at 4 °C with primary antibodies against PCNA (1:1000, Abclonal, #A0264), Caspase-9 (1:200, Proteintech, 10380-1-AP), Bax (1:1000, Abmart Shanghai Co.,Ltd, T40051), PKM2 (1:200, Proteintech, 15822-1-AP), and LDHA (1:1000, CST, C28H7). Antibody specificity was validated by the manufacturers and confirmed by the presence of single bands at the expected molecular weights in Western blot analysis (as reported in product datasheets). After washing, sections were incubated with HRP-conjugated secondary antibodies and visualized with DAB substrate. Images were captured using a microscope, and positive staining was analyzed with ImageJ software.

#### TUNEL assay

Apoptosis in ovarian sections was detected using a TUNEL apoptosis detection kit (Elabscience, #E-CK-A321) following the manufacturer’s protocol. Briefly, sections were treated with proteinase K, incubated with TUNEL reaction mixture, and then with converter-POD. DAB was used for color development, and hematoxylin was used for counterstaining. TUNEL-positive (apoptotic) cells in follicles were counted.

#### Quantitative real-time PCR (qRT-PCR)

Total RNA was extracted from ovarian tissues using TRIzol reagent (Takara, #9109). RNA quality was checked, and 1 μg RNA was reverse-transcribed into cDNA using the PrimeScript™ RT reagent Kit (Takara, #RR047A). qPCR was performed on a QuantStudio 5 system (Applied Biosystems) using TB Green® Premix Ex Taq™ II (Takara, #RR820A). Gene expression was normalized to Gapdh and calculated using the 2^(-ΔΔCt) method. Primer sequences are listed in Supplementary Table S1.

### Immunofluorescence (IF)

Ovarian sections were processed similarly to IHC. After antigen retrieval and blocking, sections were co-incubated with primary antibodies against FOXO3a (1:200, Proteintech, 66428-1- Ig) and DDX4/VASA (1:200, Proteintech, 51042-1-AP); or p-rps6 (1:1000, Proteintech, 67898-1- Ig) and rps6 (1:200, Proteintech, 14823-1-AP); or GLUT4 (1:200, Proteintech, 66846-1-Ig) and PKM2 (1:200, Proteintech, 15822-1-AP). After washing, sections were incubated with Alexa Fluor 488--conjugated (1:200, Proteintech, SA00013-1) or 594-conjugated (1:200, Proteintech, SA00013-4) secondary antibodies and counterstained with DAPI. Images were acquired using a laser scanning confocal microscope (ZEISS LSM880) by investigators blinded to group allocation.

### Gut microbiota analysis

Fresh fecal samples were collected, immediately snap-frozen in liquid nitrogen, and stored at −80 °C until further processing. A total of eight biological samples per group were analyzed. Microbial genomic DNA was extracted using the QIAamp DNA Stool Mini Kit (Qiagen, Hilden, Germany) following the manufacturer’s instructions. The V3–V4 hypervariable region of the bacterial 16S rRNA gene was amplified using the primer pair 341F (5′-CCTACGGGNGGCWGCAG-3′) and 805R (5′-GACTACHVGGGTATCTAATCC-3′) with barcoded adapters for multiplexing. Paired-end sequencing (2 × 300 bp) was performed on an Illumina MiSeq platform, aiming for a sequencing depth of at least 50,000 high-quality reads per sample. Raw sequencing data were demultiplexed, quality-filtered (truncated at positions with average quality scores < Q20 over a 10-bp sliding window), and denoised using the DADA2 plugin within QIIME2 (version 2023.9). Samples were rarefied to an even depth based on the minimum sequencing depth to normalize for differences in sequencing depth across samples. Amplicon sequence variants (ASVs) were taxonomically assigned using the SILVA reference database (release 138) with a confidence threshold of 0.7. Alpha diversity indices (Shannon, Chao1, ACE, Simpson) were compared among groups using the Kruskal-Wallis test followed by Dunn’s *post hoc* test. Beta diversity was assessed using Principal Coordinates Analysis (PCoA) and Non-metric Multidimensional Scaling (NMDS) based on Bray-Curtis distances, with group differences evaluated by PERMANOVA (999 permutations). Differential abundance analysis was performed using Linear Discriminant Analysis Effect Size (LEfSe) and the Kruskal-Wallis test, with false discovery rate (FDR) correction applied for multiple comparisons.

### *In Vitro* cell experiments

The human ovarian granulosa-like tumor cell line (KGN) was cultured. Cells were pretreated with or without the DOP preparation (200 μg/mL) for 24 h, then exposed to 5 Gy X-ray irradiation. The concentration of 200 μg/mL was selected based on previously published studies using DOP in cell culture models (Feng et al., 2022), as well as preliminary observations from our laboratory indicating that this concentration showed consistent effects without cytotoxicity (data not shown). A formal dose-response analysis was not performed to establish this as the optimal concentration. Apoptosis was assessed 24 h post-irradiation using Annexin V-FITC/PI staining and flow cytometry (Beckman CytoFLEX). Intracellular ROS levels were measured using the DCFH-DA probe and flow cytometry. Mitochondrial membrane potential (ΔΨm) was evaluated using the JC-1 assay kit (Beyotime, C2006). All *in vitro* experiments included DOP-only control groups (cells treated with DOP without irradiation) to evaluate the baseline effects of the DOP preparation on KGN cells.

### Western blot analysis

Ovarian tissues were lysed in RIPA buffer containing protease and phosphatase inhibitors. Protein concentrations were determined using the BCA assay. Equal amounts of protein (30 μg) were separated by SDS-PAGE and transferred to PVDF membranes. Membranes were blocked with 5% non-fat milk and incubated overnight at 4 °C with primary antibodies against PI3K (1:1000, Proteintech, 20584-1-AP), p-AKT (1:1000, CST, #4060), AKT (1:1000, Proteintech, 10176-2-AP), p-mTOR (1:1000, Proteintech, 67778-1-Ig), mTOR (1:1000, Proteintech, 20657-1-AP), p-FOXO3a (1:1000, CST, #9466), FOXO3a (1:1000, Proteintech, 66428-1-Ig), p-rps6 (1:1000, Proteintech, 67898-1-Ig), rps6 (1:1000, Proteintech, 14823-1-AP), and GAPDH (1:10000, Proteintech, 60004-1-Ig). After washing, membranes were incubated with HRP-conjugated secondary antibodies. Protein bands were visualized using enhanced chemiluminescence (ECL) and quantified by densitometry using ImageJ software. Phosphorylation data were normalized to the corresponding total protein levels, and all protein levels were normalized to GAPDH. A total of six independent biological samples (from six different animals per group) were analyzed. The same animals used for other ovarian analyses were used for Western blotting. Full uncropped blots and molecular-weight markers are provided in Supplementary Figure S1, and we have requested a 1-month extension to prepare these data as some original blots were previously trimmed for presentation.

### Statistical analysis

Data are presented as mean ± standard deviation (SD). Normality was assessed using the Shapiro-Wilk test, and homogeneity of variances was verified using Levene’s test before applying parametric tests. Statistical analysis was performed using SPSS 22.0 and GraphPad Prism 9.0. Differences among multiple groups were analyzed by one-way ANOVA followed by Tukey’s *post hoc* test when assumptions of normality and homogeneity of variances were met; otherwise, the Kruskal-Wallis test with Dunn’s *post hoc* test was used. For estrous cycle data, proportions of days in each stage were compared among groups using the Kruskal-Wallis test with Dunn’s *post hoc* test. For microbiota β-diversity, PERMANOVA was used. For differential abundance analysis of gut microbiota, FDR correction was applied for multiple comparisons. *P*-value < 0.05 was considered statistically significant.

## Results

### Characterization of DOP

HPLC-GPC analysis revealed that the extracted DOP preparation had an average molecular weight of 317.8 kDa (Figure 1). Monosaccharide composition analysis indicated it was primarily composed of mannose (71.43%) and glucose (26.49%), with trace amounts of galacturonic acid (2.08%) (Figures 2A,B). The polysaccharide content was 59.09%, indicating that the preparation also contained other unidentified non-polysaccharide components.

**FIGURE 1:**
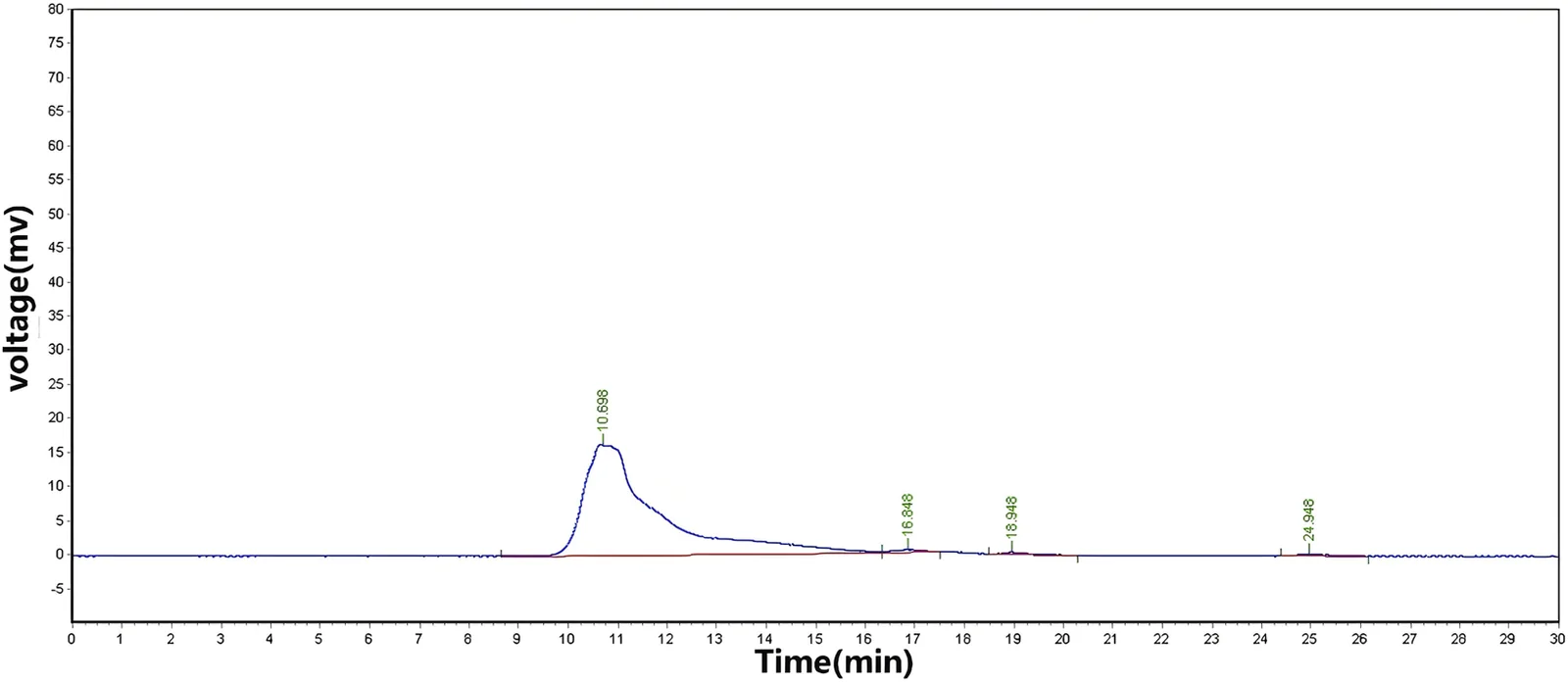
Molecular weight determination of *Dendrobium officinale* polysaccharides (DOP) by high-performance liquid chromatography (HPLC). The retention volume (Ve) of DOP is 10.698 mL. The partition coefficient (Kav) was calculated using the standard formula Kav = (Ve - V0)/(Vt - V0), where V0 (void volume) = 7.15 mL and Vt (total volume) = 19.45 mL, yielding Kav = 0.315. The logarithmic molecular weight (lgMw) was then derived from the standard curve equation y = −3.3056x + 5.5021 (where x = Kav), giving lgMw = 4.461. Consequently, the molecular weight of DOP is calculated as 2.89 × 10^4^ Da.

**FIGURE 2:**
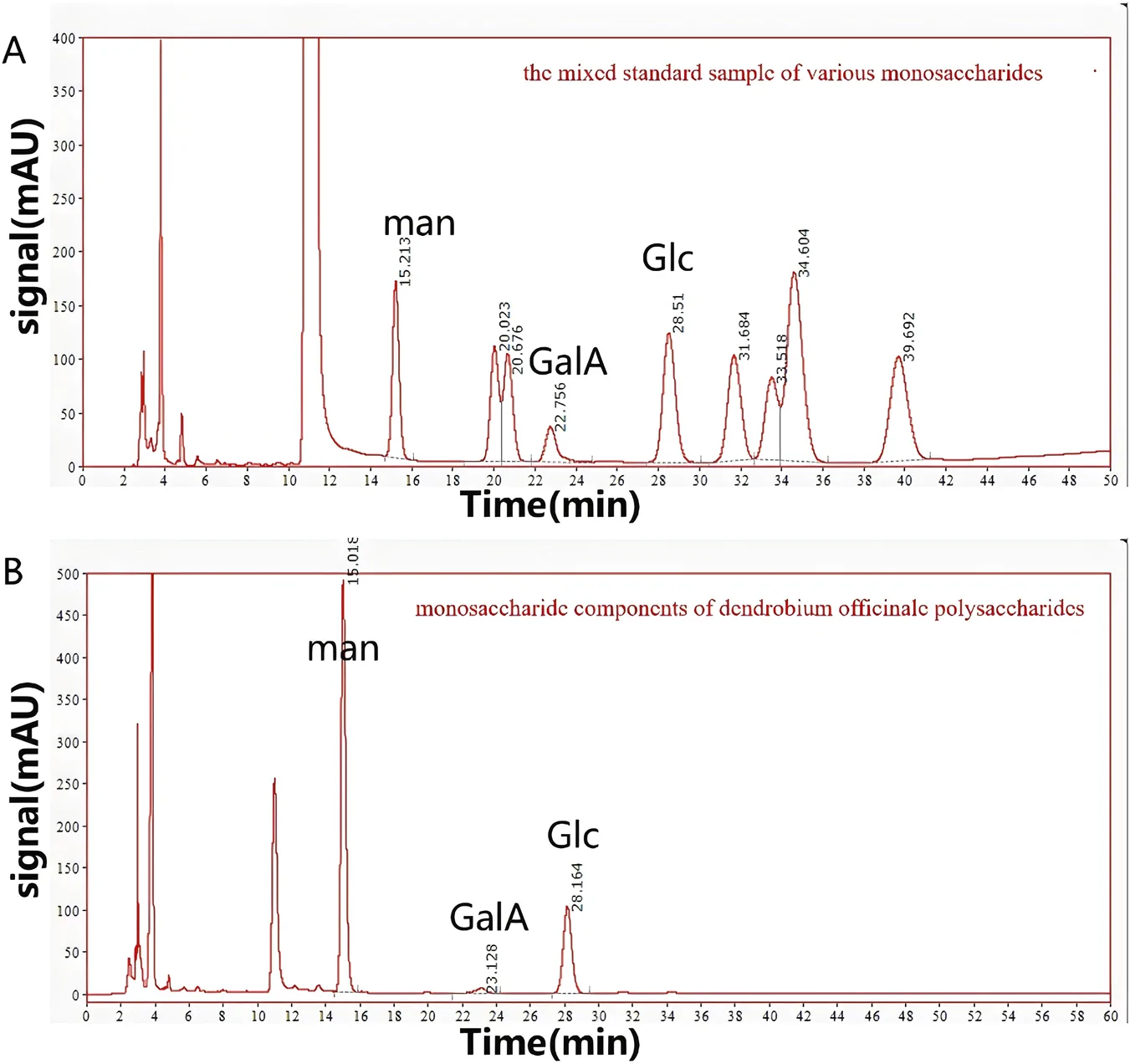
Monosaccharide composition analysis of DOP by HPLC. **(A)** HPLC chromatogram of monosaccharide standards. **(B)** HPLC chromatogram of DOP hydrolysate. The molar ratio of Mannose (Man): Galacturonic acid (GalA): Glucose (Glc) was determined to be 34.40: 1: 12.76. The polysaccharide content was 59.09%, with an average molecular weight of 317.8 kDa. The mass percentage composition was: Man, 71.43%; GalA, 2.08%; Glc, 26.49%.

### DOP ameliorates systemic and ovarian phenotypes in irradiated mice

Irradiation caused a transient decrease in food intake within the first 5 days, but no significant change in final body weight among groups (Figures 3A,B). Although the transient reduction in food intake during the early post-irradiation period could have influenced metabolism or gut microbiota, the absence of a significant difference in final body weight suggests that nutritional status was largely restored. However, irradiated mice (5 Gy group) exhibited severe estrous cycle disruption (prolonged diestrus or acyclicity), which was significantly improved by DOP pretreatment (Figures 3C,D). Consistent with a POI phenotype, the 5 Gy group showed significantly elevated serum FSH and LH levels and markedly reduced E2 and AMH levels compared to the control group. DOP intervention effectively reversed these hormonal imbalances (Figures 4A–D). Furthermore, irradiation led to a significant reduction in ovarian and uterine indices, indicative of gonadal atrophy. DOP pretreatment largely preserved the size and weight of these organs (Figures 5A–D). H&E staining confirmed that DOP attenuated radiation-induced follicular depletion, increasing the numbers of primordial and growing follicles while decreasing the proportion of atretic follicles (Figures 5E,F). DOP also ameliorated irradiation-induced endometrial thinning and glandular atrophy (Figure 5G).

**FIGURE 3:**
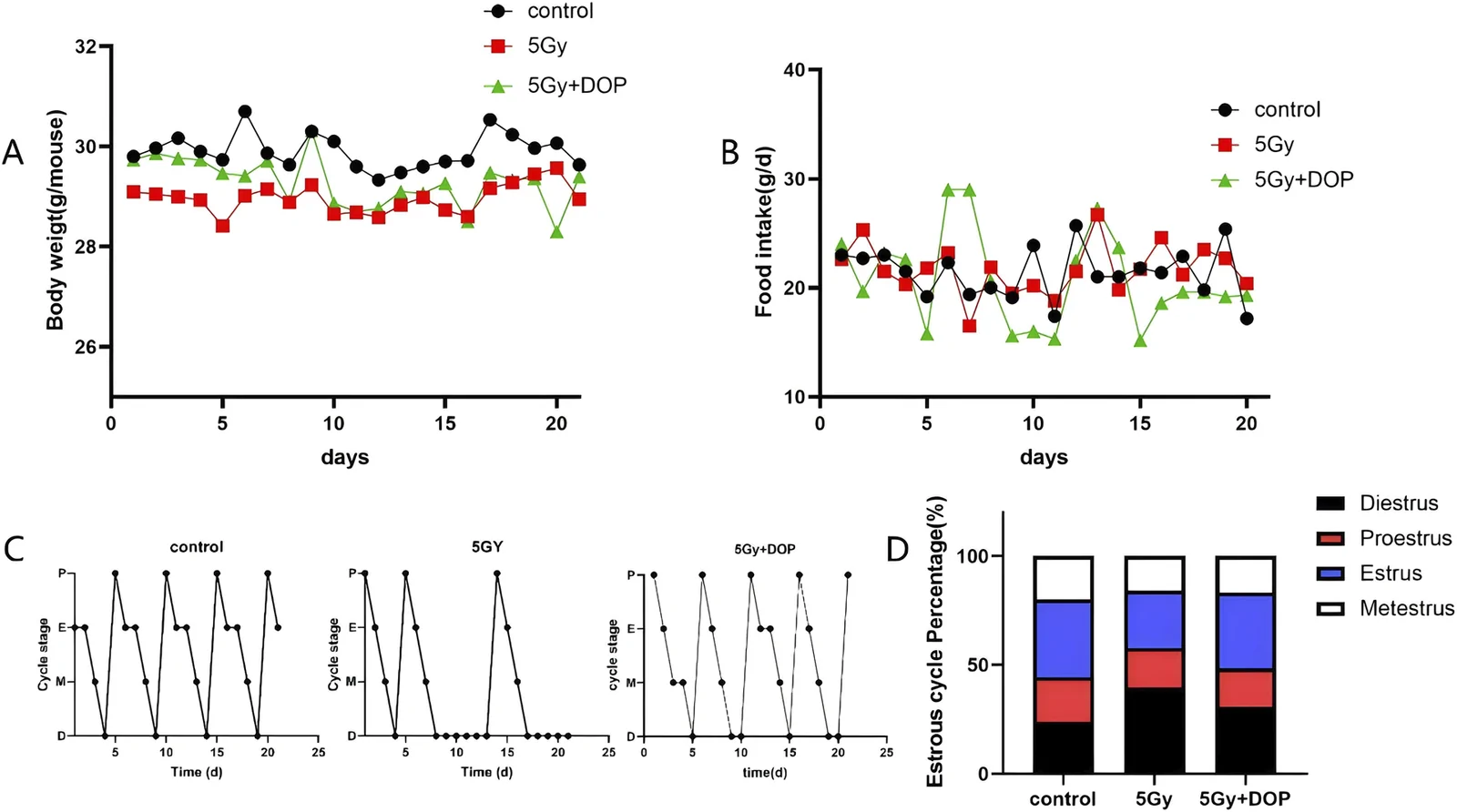
DOP intervention ameliorates systemic and estrous cycle disturbances in irradiated mice. **(A)** Body weight changes of mice in different groups over time. **(B)** Daily food intake of mice post-irradiation. **(C)** Representative cytology images depicting the estrous cycle stages (Proestrus, Estrus, Metestrus, Diestrus) for each group. **(D)** Quantitative distribution of estrous cycle stages among groups over the 14-day monitoring period. Data are presented as mean ± SD (n = 10). For estrous cycle data, the proportion of days in each stage was compared using the Kruskal-Wallis test with Dunn’s *post hoc* test. P < 0.05, P < 0.01 vs. Control group. #P* < 0.05, *P* < 0.01 vs. 5 Gy group.

**FIGURE 4:**
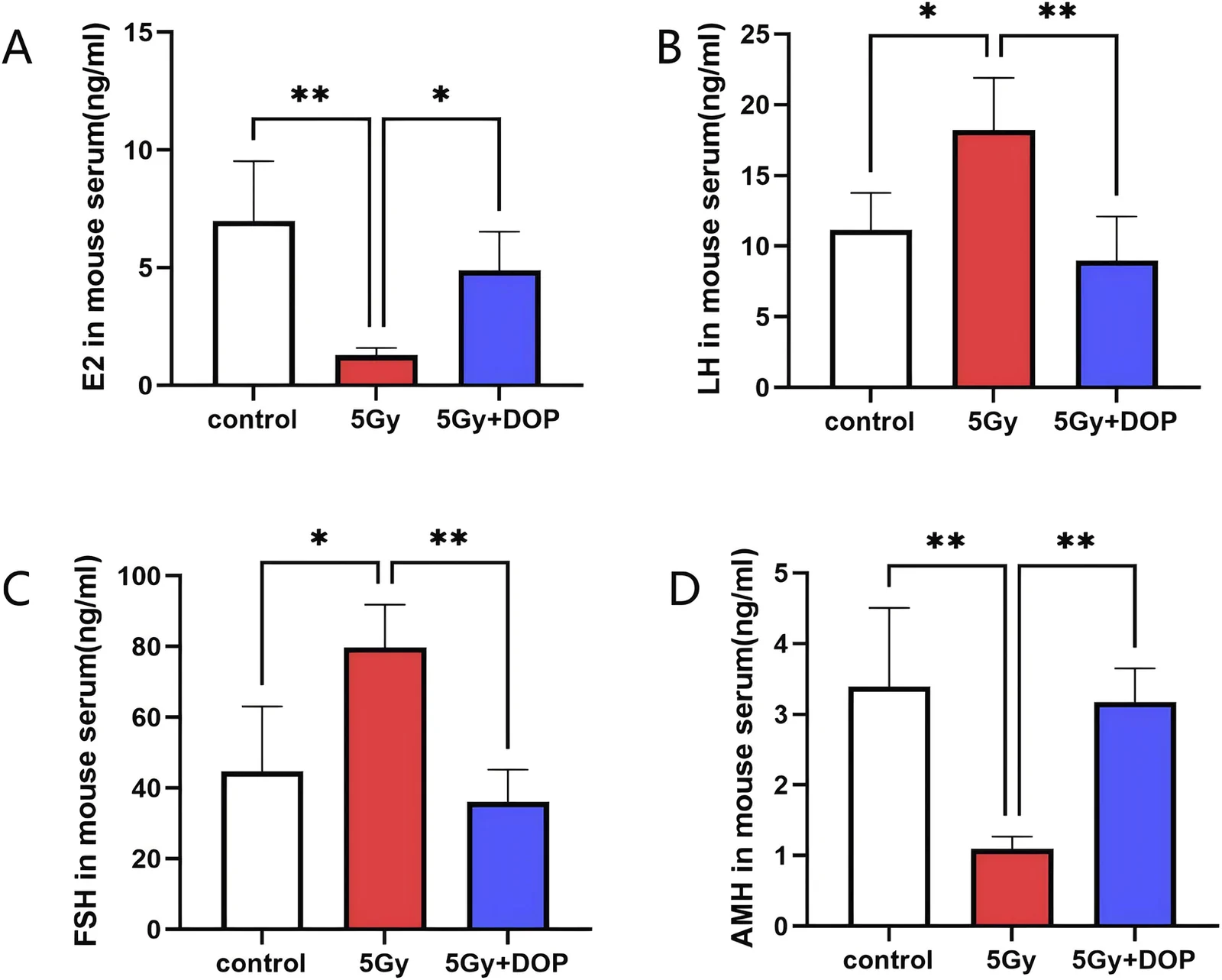
DOP restores serum sex hormone levels in mice with radiation-induced POI. Serum concentrations of **(A)** estradiol (E2), **(B)** luteinizing hormone (LH), **(C)** follicle-stimulating hormone (FSH), and **(D)** anti-Müllerian hormone (AMH) were measured by ELISA at day 14 post-irradiation. Data are presented as mean ± SD (n = 10). One-way ANOVA with Tukey’s *post hoc* test was used. *P* < 0.05, *P < 0.01 vs. Control group; *P* < 0.05, *P < 0.01 vs. 5 Gy group.

**FIGURE 5:**
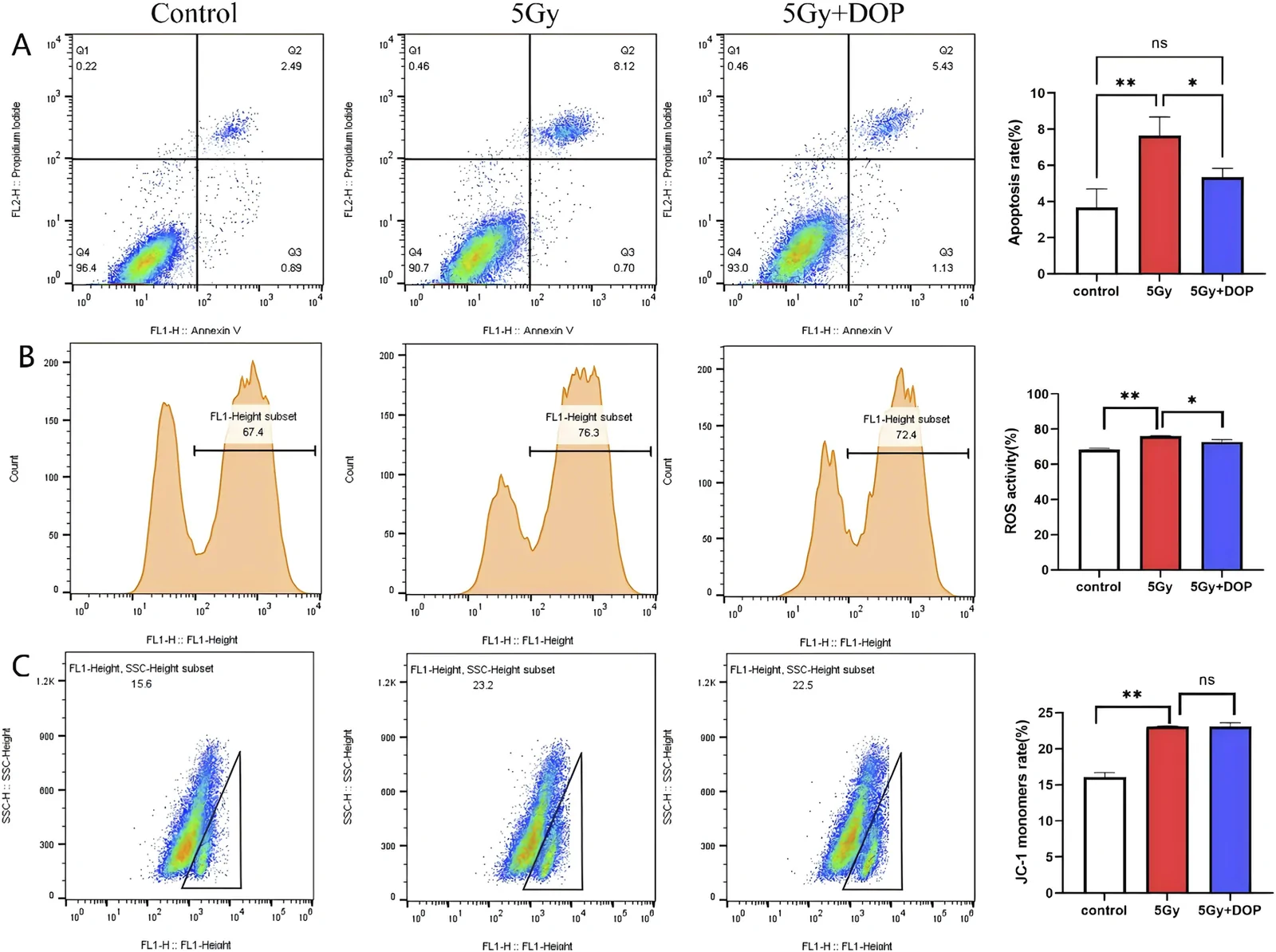
DOP pretreatment preserves ovarian and uterine morphology and follicular reserve in POI mice. **(A)** Gross morphological appearance of ovaries. **(B)** Gross morphological appearance of uteri. **(C)** Ovarian index (ovary weight/body weight × 100%). **(D)** Uterine index (uterus weight/body weight × 100%). **(E)** Quantitative analysis of follicle counts at different developmental stages. **(F)** Representative H&E-stained sections of ovarian tissue (scale bar, 200 µm). **(G)** Representative H&E-stained sections of uterine tissue (scale bar, 200 µm). Data in C-E are mean ± SD (n = 10). One-way ANOVA with Tukey’s *post hoc* test was used. *P* < 0.05, *P < 0.01 vs. Control; *P* < 0.05, *P < 0.01 vs. 5 Gy. PF: primordial follicle; GF: growing follicle; AF: antral follicle; AtrF: atretic follicle.

### DOP suppresses radiation-induced apoptosis in granulosa cells

To evaluate granulosa cell apoptosis, we examined the expression of pro-apoptotic proteins and performed TUNEL staining. IHC analysis showed that the pro-apoptotic proteins Bax and Cleaved Caspase-9 were significantly upregulated in the ovaries of the 5 Gy group compared to the Control group, an effect markedly mitigated by DOP pretreatment (Figure 6A). The TUNEL assay further corroborated these findings, revealing a significant increase in apoptotic granulosa cells within atretic follicles of the 5 Gy group, which was substantially reduced in the DOP-treated group (Figure 6B). *In vitro* experiments using KGN cells were conducted to validate the anti-apoptotic effects of DOP. Flow cytometry analysis demonstrated that DOP pretreatment significantly decreased the apoptosis rate in irradiated KGN cells (Figure 7A). Additionally, DOP effectively lowered intracellular reactive oxygen species (ROS) levels, as measured by the DCFH-DA probe (Figure 7B). DOP did not significantly restore mitochondrial membrane potential (ΔΨm) under the present experimental conditions (*P* > 0.05) (Figure 7C). Therefore, these data do not support preservation of mitochondrial membrane potential as a major mechanism of DOP action.

**FIGURE 6:**
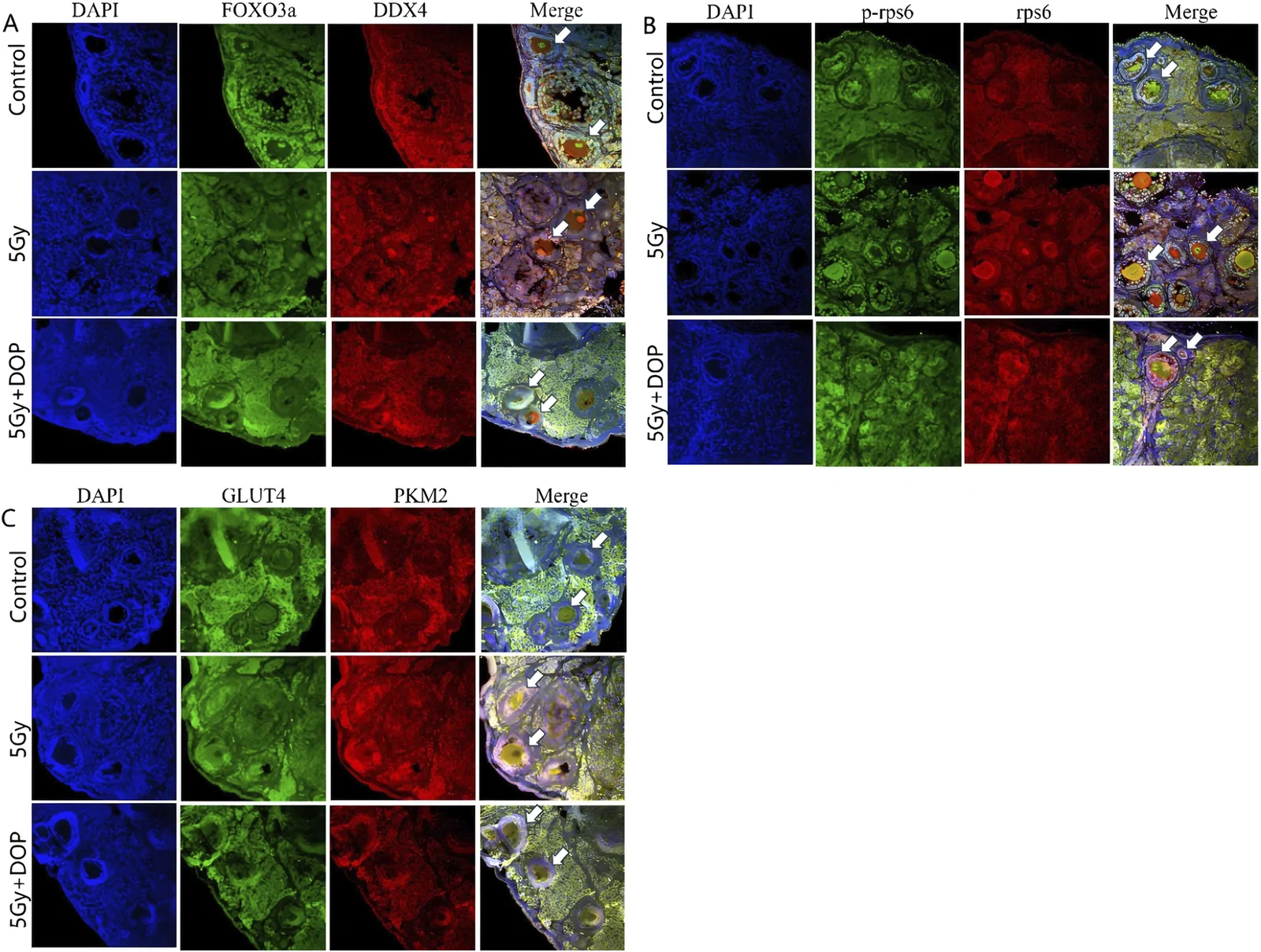
DOP inhibits radiation-induced apoptosis in ovarian tissue. **(A)** Representative IHC images showing the expression of pro-apoptotic proteins Bax and Caspase-9 in ovarian sections (scale bar, 50 µm). **(B)** Detection of apoptotic cells in ovarian tissue by TUNEL assay (scale bar, 100 µm). Data are representative of three independent experiments (n = 6 per group).

**FIGURE 7:**
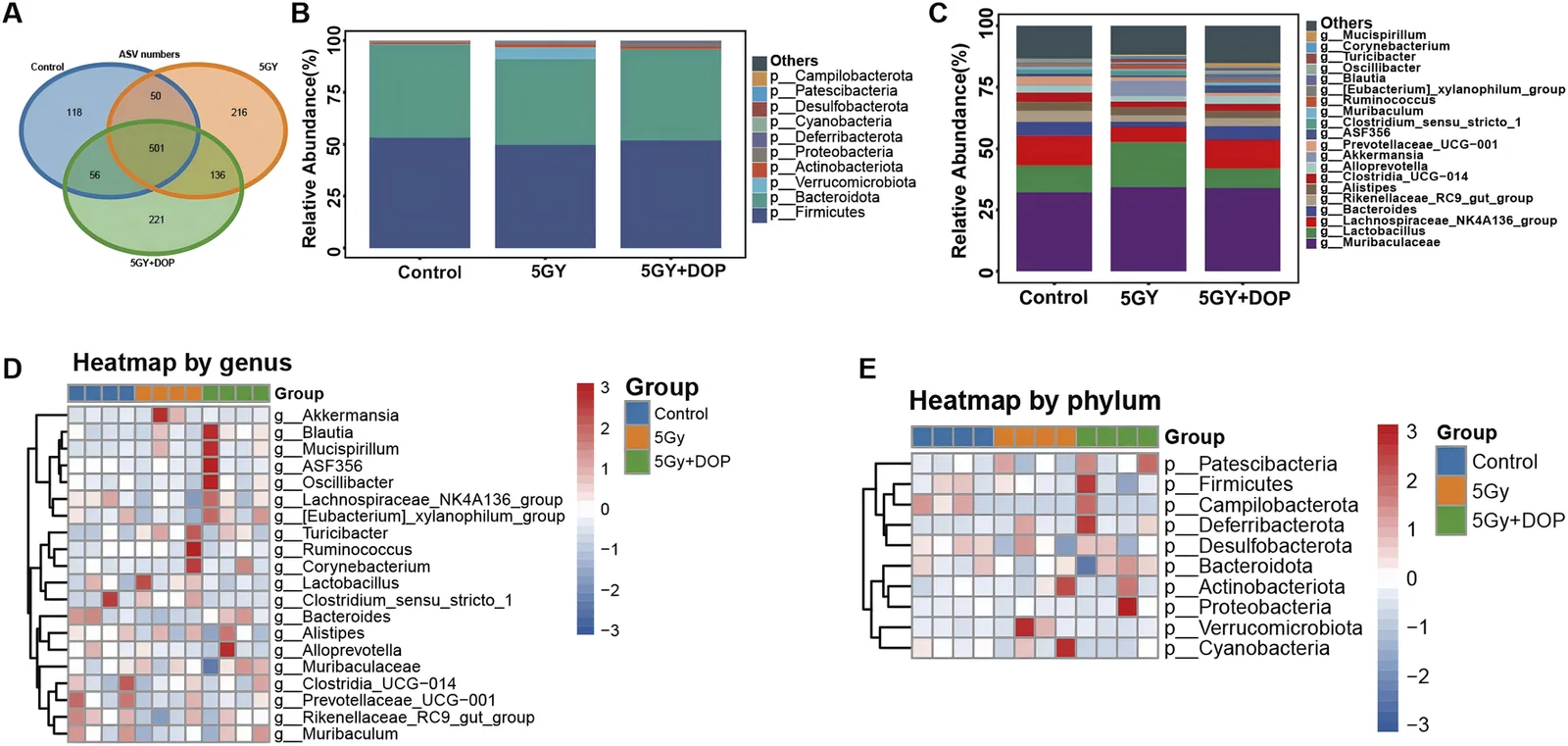
DOP mitigates X-ray-induced damage in human ovarian granulosa (KGN) cells. **(A)** Apoptosis rates were assessed by flow cytometry with Annexin V-FITC/PI staining. **(B)** Intracellular reactive oxygen species (ROS) levels were measured using the DCFH-DA probe. **(C)** Mitochondrial membrane potential (ΔΨm) was evaluated using the JC-1 assay; the ratio of red (aggregated JC-1) to green (monomeric JC-1) fluorescence indicates ΔΨm. Data are presented as mean ± SD from three independent experiments. One-way ANOVA with Tukey’s post hoc test was used. P < 0.05, P < 0.01 vs. Control; P < 0.05, P < 0.01 vs. 5 Gy. DOP did not significantly restore mitochondrial membrane potential under the present experimental conditions (P > 0.05).

#### DOP modulates the PI3K/AKT signaling pathway in a follicle stage-specific manner

Given the central role of PI3K/AKT in follicle fate, we investigated its status. Immunofluorescence analysis of primordial follicles at an early time point (3 days post-irradiation) revealed that in the 5 Gy group, primordial follicles exhibited decreased nuclear FOXO3a fluorescence (indicating inactivation/export) and increased p-rps6 fluorescence, signifying overactivation of the PI3K/AKT pathway specifically in this dormant pool. DOP treatment reversed these changes, promoting FOXO3a nuclear retention and reducing p-rps6 signal in primordial follicles (Figures 8A,B). These data provide evidence of altered FOXO3a localization and p-rps6 signaling specifically in primordial follicles. However, comparable quantitative analyses for primary, secondary, and other growing follicles were not performed; therefore, the model of “follicle stage-specific regulation” across all follicle stages remains to be fully established. Consistent with these findings, qPCR analysis showed that DOP treatment restored the mRNA expression levels of key pathway components, including PI3K, AKT, mTOR, FOXO3a, and rps6 (Figure 8C). These findings suggest that DOP inhibits radiation-induced premature activation of primordial follicles while supporting survival signaling in growing follicles.

**FIGURE 8:**
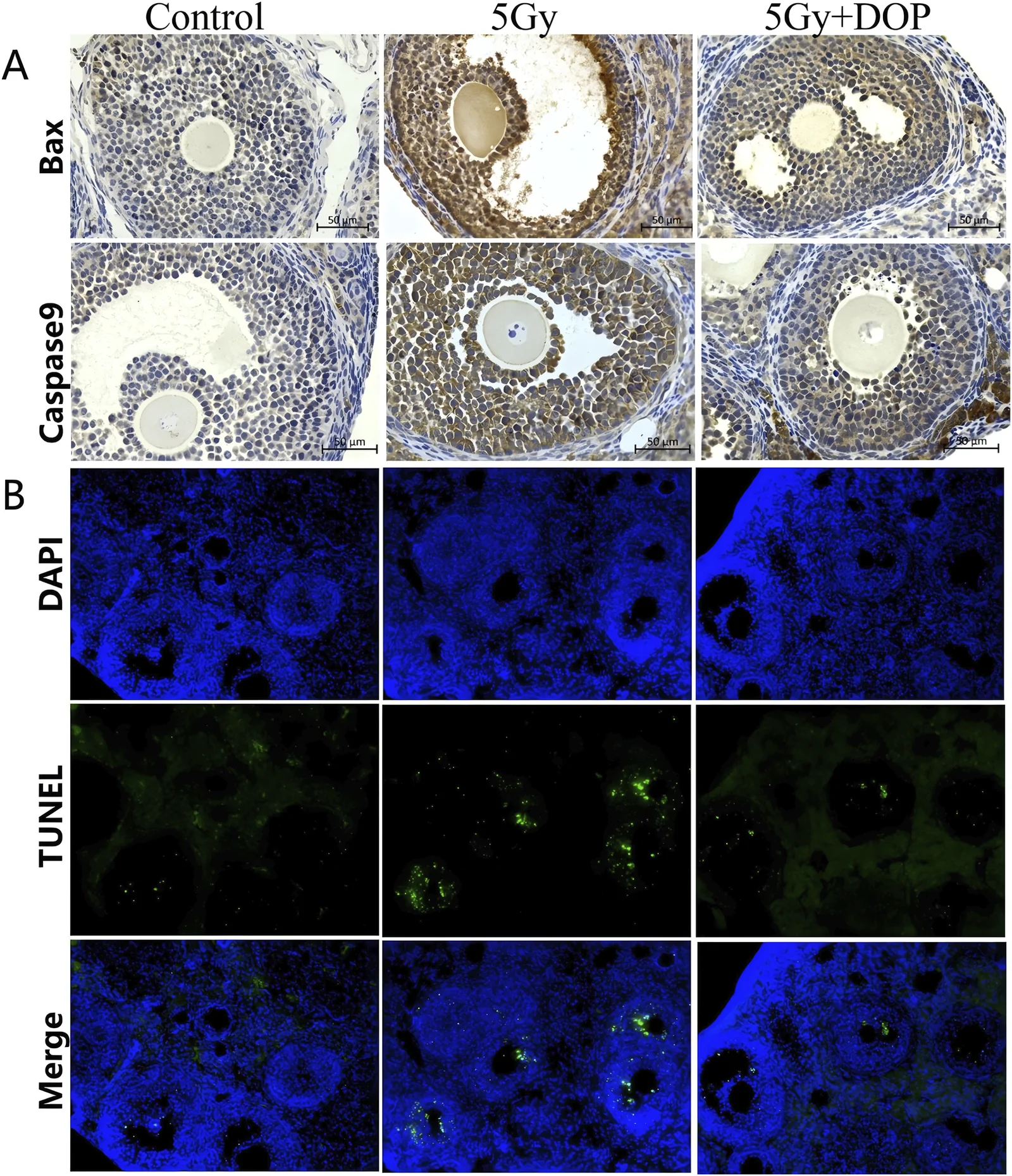
DOP regulates PI3K/AKT pathway activity and glycolysis in ovarian follicles. **(A,B)** Immunofluorescence (IF) staining of ovarian sections (scale bar, 20 µm) at 3 days post-irradiation. **(A)** Co-staining of FOXO3a (green) and the primordial follicle marker DDX4 (red). Nuclei are counterstained with DAPI (blue). FOXO3a nuclear retention (merged yellow in nuclei) indicates pathway suppression. **(B)** Co-staining of phosphorylated rps6 (p-rps6, green) and total rps6 (red). Increased green signal indicates PI3K/AKT/mTOR pathway activation. **(C)** Relative mRNA expression levels of PI3K, AKT, mTOR, FOXO3a, and rps6 determined by qRT-PCR. **(D)** Co-staining of the glucose transporter GLUT4 (green) and the glycolytic enzyme PKM2 (red). Arrows indicate representative primordial follicles. Data in C are mean ± SD (n = 6). One-way ANOVA with Tukey’s *post hoc* test was used. *P < 0.05, *P* < 0.01 vs. Control group; *P < 0.05, *P* < 0.01 vs. 5 Gy group; ns, not significant.

To further elucidate the molecular mechanism underlying DOP mediated ovarian protection, we examined the status of the PI3K/AKT signaling pathway, a central regulator of follicle survival and activation. Western blot analysis was performed using six independent biological samples per group (from the same animals used for other ovarian analyses). Phosphorylation data were normalized to the corresponding total protein levels, and all protein levels were normalized to GAPDH. Full uncropped Western blot images with molecular-weight markers are provided in Supplementary Figure S1. Western blot analysis revealed that total protein levels of PI3K, AKT, mTOR, FOXO3a, and rps6 remained comparable across all three groups. However, irradiation with 5 Gy X-ray significantly reduced the phosphorylation levels of AKT (p-AKT), mTOR (p-mTOR), FOXO3a (p-FOXO3a), and rps6 (p-rps6) in ovarian tissues compared to the Control group, indicating a marked suppression of the PI3K/AKT/mTOR signaling cascade. Notably, DOP pretreatment effectively reversed this radiation-induced suppression, as evidenced by a significant upregulation of all four phosphorylated proteins in the 5 Gy + DOP group relative to the 5 Gy group (Figures 9A,B). Original, uncropped membrane images corresponding to the blots shown in Figure 9 are available in Supplementary Figure S1. While these data demonstrate restoration or increased phosphorylation of pathway-related proteins in DOP-treated ovarian tissues, they do not by themselves establish that this pathway is the exclusive mediator of ovarian protection. Consistent with these protein-level findings, qRT-PCR analysis demonstrated that the mRNA expression of PI3K, AKT, mTOR, FOXO3a, and rps6 exhibited similar trends across groups, with irradiation downregulating their transcript levels and DOP treatment restoring them toward control values (Figure 9C). Collectively, these results suggest that DOP counteracts radiation-induced ovarian injury, at least in part, by modulation the PI3K/AKT signaling pathway at both transcriptional and post-translational levels.

**FIGURE 9:**
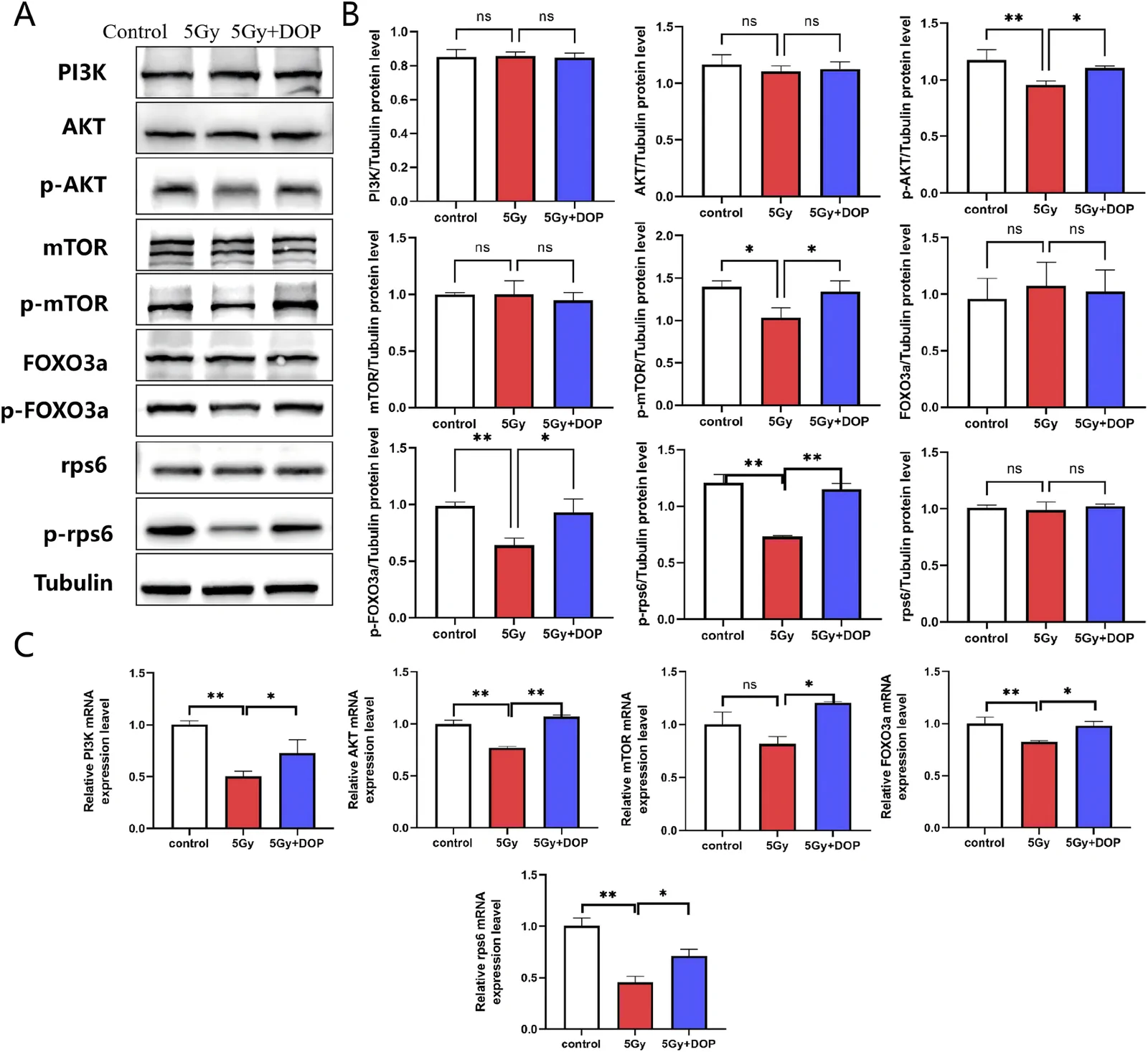
DOP modulates the PI3K/AKT signaling pathway in the ovaries of POI mice. **(A)** Representative Western blot images showing the protein expression levels of PI3K/AKT pathway-related components, including PI3K, p-AKT, AKT, p-mTOR, mTOR, p-FOXO3a, FOXO3a, p-rps6, and rps6, in ovarian tissues. The full-length, uncropped Western blot images with molecular-weight markers are provided in Supplementary Figure S1. **(B)** Densitometric quantification of protein levels normalized to their respective total proteins. **(C)** Relative mRNA expression levels of PI3K, AKT, mTOR, FOXO3a, and rps6 determined by qRT-PCR. Data are presented as mean ± SD (n = 6 independent biological samples per group). One-way ANOVA with Tukey’s *post hoc* test was used. P < 0.05, P < 0.01 vs. Control group; *P* < 0.05, P* < 0.01 vs. 5 Gy group; ns, not significant.

### DOP restores gut microbiota homeostasis disrupted by irradiation

Histological examination of colonic tissues revealed that irradiation induced marked mucosal damage, characterized by glandular atrophy, architectural disorganization, and inflammatory cell infiltration, all of which were substantially ameliorated by DOP pretreatment (Figure 10A). 16S rRNA sequencing of eight biological samples per group (no samples failed quality-control criteria) further demonstrated that irradiation significantly reduced gut microbial α-diversity, as reflected by decreased Shannon and Chao1 indices (Kruskal-Wallis test with Dunn’s *post hoc* test, FDR-corrected), whereas DOP treatment effectively restored these diversity measures (Figures 10B,C). β-Diversity analysis via PCoA and NMDS based on Bray-Curtis distances (PERMANOVA, 999 permutations) showed that the microbial community structure of the 5 Gy group diverged distinctly from both the Control and 5 Gy + DOP groups, with the latter two exhibiting close clustering, indicating that DOP treatment reshaped the irradiated gut microbiota toward a composition resembling that of healthy controls (Figure 10D). At the phylum level, irradiation lowered the Firmicutes/Bacteroidetes (F/B) ratio and elevated the relative abundance of Verrucomicrobia; these shifts were largely normalized by DOP administration (Figures 11A,E). At the genus level, DOP enriched potentially beneficial taxa, including Muribaculum and Lachnospira, while suppressing several genera that expanded in response to irradiation (Figures 11B–D).

**FIGURE 10:**
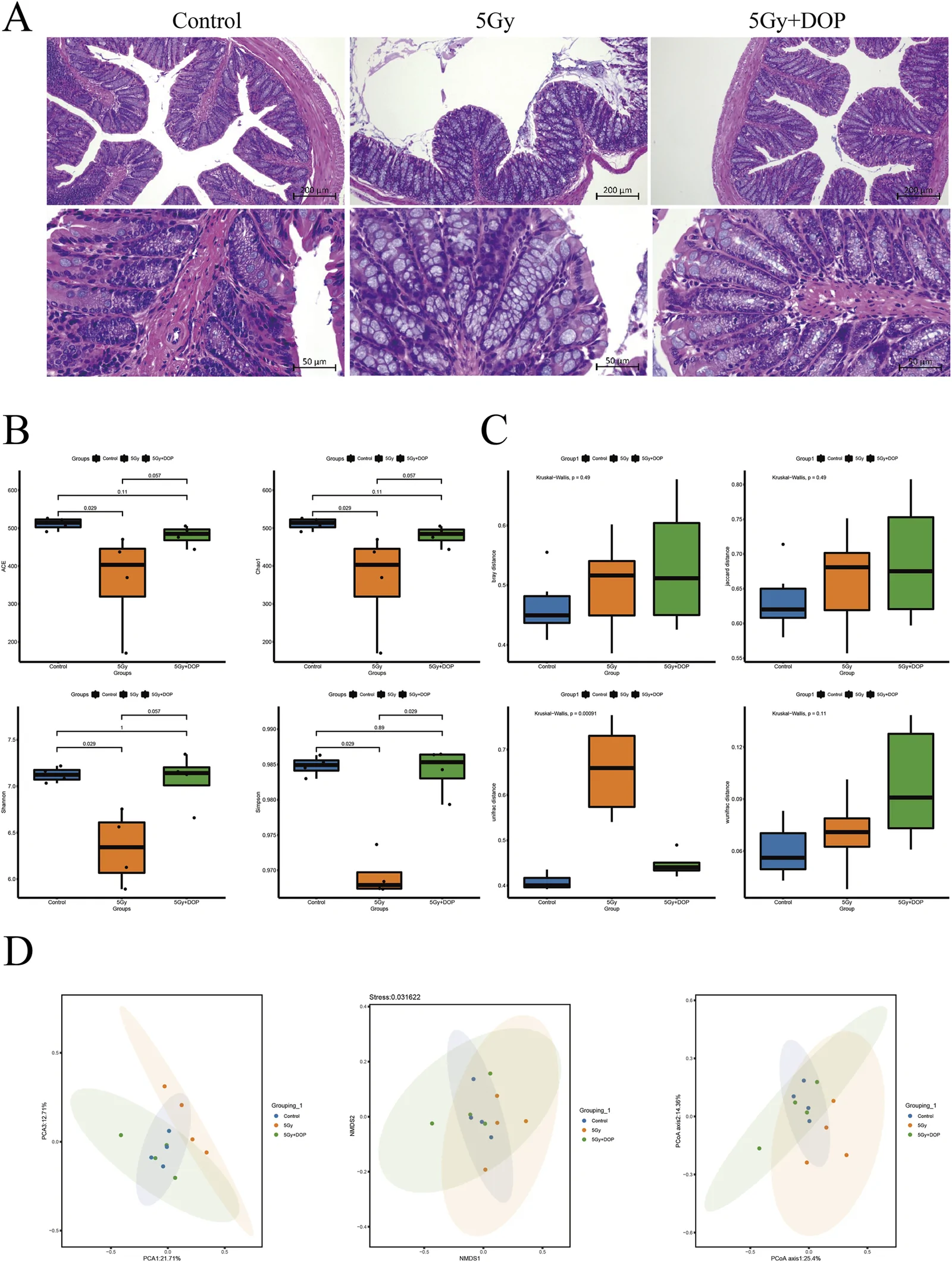
DOP ameliorates radiation-induced colonic mucosal damage and restores gut microbiota diversity. **(A)** Representative H&E-stained sections of mouse colon tissues (scale bars: upper panel, 200 µm for ×10 objective; lower panel, 50 µm for ×40 objective). The 5 Gy group exhibits severe mucosal damage characterized by glandular atrophy, disorganized architecture, inflammatory cell infiltration, and increased mucus production. DOP pretreatment markedly preserved the colonic mucosal structure, making it comparable to the Control group. **(B)** α-Diversity indices (Chao1, ACE, Shannon, Simpson) of gut microbial communities, reflecting species richness and evenness. **(C)** Box plots showing within-group β-diversity distances calculated using Bray-Curtis, Jaccard, unweighted UniFrac, and weighted UniFrac metrics. These distances assess sample homogeneity; smaller boxes indicate higher intra-group similarity. **(D)** β-Diversity analysis visualized by Principal Component Analysis (PCA, PC1 vs PC3), Non-metric Multidimensional Scaling (NMDS, MDS1 vs MDS2), and Principal Coordinates Analysis (PCoA, axis1 vs axis2), with the latter two based on Bray-Curtis distances. Each point represents a sample; closer points indicate more similar microbial communities. Data are presented as mean ± SD (n = 8 biological samples per group). For α-diversity, the Kruskal-Wallis test with Dunn’s *post hoc* test was used. For β-diversity, PERMANOVA (999 permutations) was used.

**FIGURE 11:**
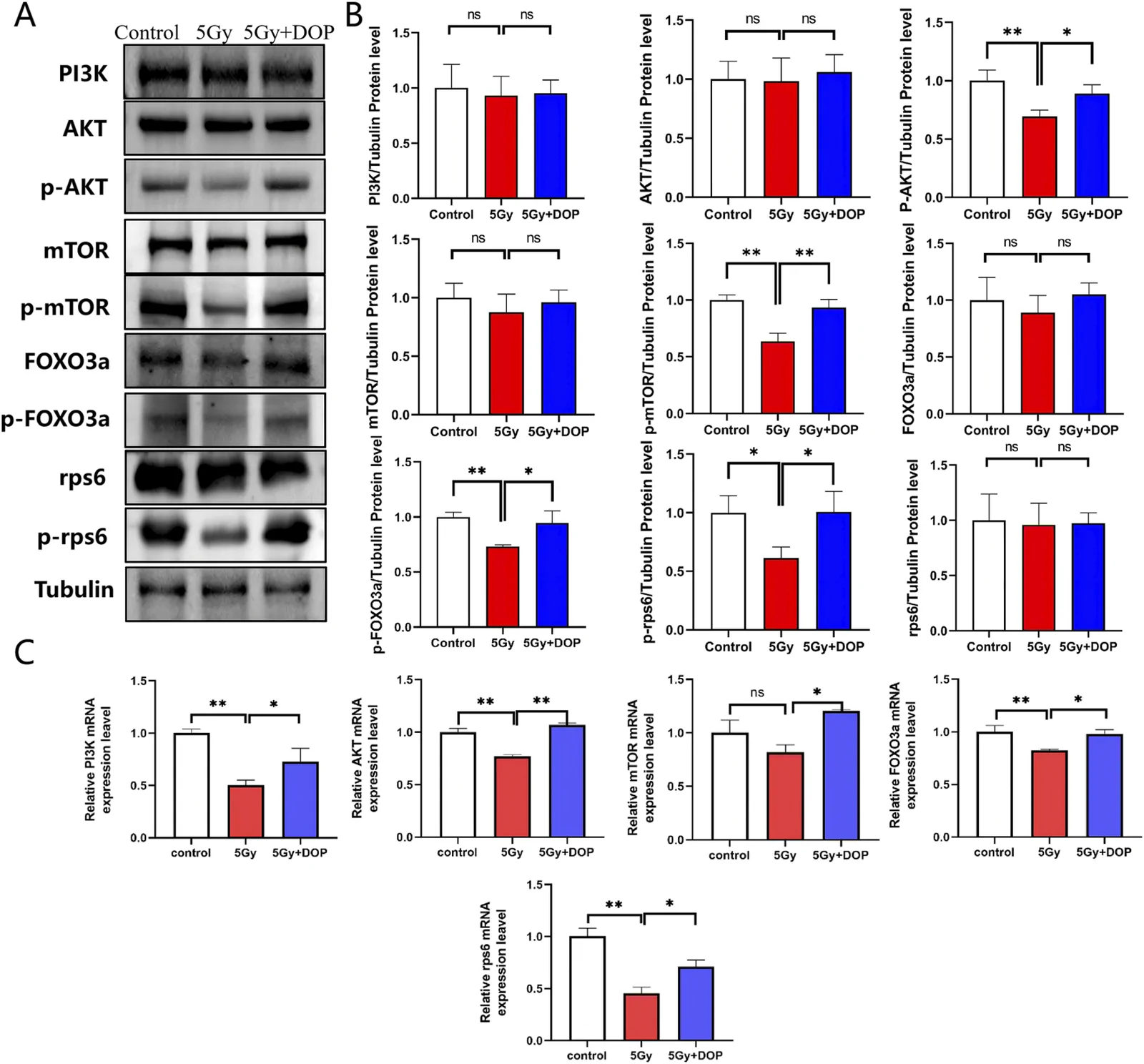
DOP reverses radiation-induced alterations in gut microbiota composition. **(A)** Venn diagram showing the number of unique and shared Amplicon Sequence Variants (ASVs) among the three groups. **(B)** Relative abundance bar plot of the dominant bacterial phyla. **(C)** Relative abundance bar plot of the top 15 bacterial genera. **(D)** Heatmap displaying the relative abundance of significantly different genera (rows) across samples (columns). Color intensity represents Z-score normalized abundance. **(E)** Heatmap displaying the relative abundance of significantly different phyla. In panels D and E, asterisks denote statistical significance between the 5 Gy and Control or 5 Gy + DOP groups (*P* < 0.05, *P < 0.01).

## Discussion

In this study, we provide comprehensive evidence that pretreatment with a partially purified DOP confers significant protection against X-ray radiation-induced premature ovarian insufficiency (POI) in mice. To our knowledge, this is the first report suggesting the radioprotective effects of a DOP preparation on the ovary. The protective effects manifest at systemic, organ, cellular, and molecular levels, suggesting involvement of mediated through a sophisticated, multi-targeted mechanism involving direct anti-apoptotic actions, stage-specific modulation of the PI3K/AKT signaling pathway in a manner suggestive of stage-specific effects, upregulation of glycolysis-related marker expression, and restoration of gut microbiota homeostasis.

The DOP preparation attenuates radiation-induced ovarian damage and preserves follicle reserve. Our results clearly show that DOP pretreatment effectively alleviates the hallmark features of POI: it restores estrous cyclicity, rectifies the aberrant serum hormone profile (low AMH/E2, high FSH/LH), and preserves ovarian morphology and the follicle reserve. The observed increase in ovarian and uterine indices, along with improved endometrial thickness, indicates a broader beneficial effect of DOP on the entire female reproductive tract. These findings align with previous studies showing that polysaccharides from Dendrobium species can ameliorate reproductive dysfunction in PCOS models (Feng et al., 2022; Hu et al., 2024). Furthermore, DOP has been shown to protect against natural aging-induced premature ovarian failure by reducing p53 expression and increasing Bcl-2 expression in the ovaries of aging mice (Wu et al., 2018). However, our study uniquely extends these observations to the context of radiation injury, a clinically relevant model of iatrogenic POI. Recent studies have explored various strategies against chemotherapy- and radiotherapy-induced ovarian damage, including stem cell-based therapies (El-Derany et al., 2021), exosome interventions (Robalo Cordeiro et al., 2024), and pharmacological agents such as quercetin (Mai et al., 2025; Wang Y. et al., 2025) and other natural compounds. For instance, a recent study using a chemotherapy-induced POI model demonstrated the protective effects of certain biologics via modulation of apoptosis and oxidative stress (Atwa et al., 2025). Although our radiation-induced model differs in etiology, the shared pathways of apoptosis and PI3K/AKT signaling suggest that DOP may offer a complementary approach to existing regenerative therapies.

The DOP preparation inhibits radiation-induced apoptosis in granulosa cells via ROS suppression. Apoptosis is the primary mechanism of radiation-induced follicle loss (Tilly and Kolesnick, 2002). Our data demonstrate that irradiation significantly increases the expression of pro-apoptotic proteins (Bax, Caspase-9) and TUNEL-positive granulosa cells, effects that were reversed by DOP treatment. DOP treatment reverses these effects. *In vitro* experiments using KGN cells confirmed that DOP reduces intracellular ROS levels and decreases apoptosis after irradiation; however, DOP did not significantly restore mitochondrial membrane potential under the present experimental conditions. Therefore, these data do not support preservation of mitochondrial membrane potential as a major mechanism of DOP action. Given that ROS are key mediators of radiation-induced cellular injury, the antioxidant properties of DOP likely contribute substantially to its protective effects (Reczek and Chandel, 2015; Zeng et al., 2017). Polysaccharides are known to scavenge free radicals directly and upregulate endogenous antioxidant enzymes, though the specific mechanisms require further investigation (Zhong et al., 2022). While we did not directly quantify oxidative stress markers such as MDA, SOD, or CAT in ovarian tissue, the reduction in intracellular ROS levels observed in DOP-treated KGN cells, combined with the known antioxidant properties of DOP (Tang et al., 2017; Zhong et al., 2022), supports the interpretation that ROS suppression contributes to the anti-apoptotic effect. Future studies incorporating tissue-level oxidative stress measurements would further strengthen this conclusion.

The DOP preparation exerts effects suggestive of follicle stage-specific modulation of the PI3K/AKT signaling pathway. One of the most intriguing findings of our study is the suggested bidirectional regulation of the PI3K/AKT pathway by DOP depending on the follicle stage. Immunofluorescence analysis showed that irradiation caused overactivation of PI3K/AKT signaling in primordial follicles, as evidenced by FOXO3a nuclear export and increased p-rps6 signal. DOP reversed these changes, promoting FOXO3a nuclear retention and reducing p-rps6 signal, thereby preserving the ovarian reserve. These data provide evidence of altered FOXO3a localization and p-rps6 signaling specifically in primordial follicles. However, comparable quantitative analyses for primary, secondary, and other growing follicles were not performed. These observations were strongly supported by Western blot analysis, which demonstrated that DOP pretreatment significantly restored the phosphorylation levels of AKT, mTOR, FOXO3a, and rps6 that were suppressed by irradiation, confirming pathway modulation at the protein level. While these data demonstrate restoration or increased phosphorylation of pathway-related proteins in DOP-treated ovarian tissues, they do not by themselves establish that this pathway is the exclusive mediator of ovarian protection. qPCR data further corroborated these findings, showing that DOP restored the mRNA expression of PI3K, AKT, mTOR, FOXO3a, and rps6. This nuanced regulatory capacity of DOP highlights its sophisticated therapeutic action and distinguishes it from non-specific pathway activators or inhibitors.

The DOP preparation modulates the expression of glycolysis-related markers in ovarian follicles. Granulosa cell glycolysis is essential for supporting oocyte development, as the oocyte itself has limited glycolytic capacity (Su et al., 2009). Our study shows that irradiation suppresses the expression of key glycolytic proteins (GLUT4, HK1, PKM2, LDHA) in the ovary. DOP treatment effectively upregulates these enzymes to near-normal levels as demonstrated by IHC, IF, and qPCR. PKM2, in particular, is a rate-limiting enzyme in glycolysis and has been implicated in regulating cell proliferation and survival (Yang and Lu, 2015). However, it should be noted that increased expression of glycolytic enzymes does not necessarily prove enhanced glycolytic function. Functional assays such as glucose uptake, lactate production, ATP measurement, or extracellular acidification rate would be required to definitively conclude enhanced glycolytic capacity. Therefore, we interpret our findings as indicating that DOP modulates the expression of glycolysis-related markers rather than directly proving enhanced glycolytic function. The DOP-mediated upregulation of glycolysis-related marker expression likely ensures an adequate energy supply for follicular development and repair, complementing the pro-survival PI3K/AKT signaling. The coupling of metabolic support and anti-apoptotic signaling is crucial for follicular resilience after injury (Sutton-McDowall et al., 2010).

The DOP preparation restores radiation-induced gut microbiota dysbiosis. A novel and significant contribution of our study is the demonstration that DOP effectively remediates gut microbiota dysbiosis caused by whole-body irradiation. Ionizing radiation is well-known to cause gastrointestinal syndrome, characterized by mucosal damage, increased intestinal permeability, and severe dysbiosis (Li et al., 2025; Wang Z. et al., 2025). Our H&E staining of colonic tissue confirmed that DOP protects the colonic mucosa from radiation-induced damage. 16S rRNA sequencing revealed that irradiation significantly reduced α-diversity (Shannon, Chao1 indices) and altered β-diversity, indicating disruption of the gut microbial ecosystem. DOP treatment not only restored diversity but also enriched beneficial bacteria such as Muribaculum and Lachnospira, which are known producers of short-chain fatty acids (SCFAs) like butyrate and acetate (Lagkouvardos et al., 2019; Meehan and Beiko, 2014).

How might restoration of gut microbiota contribute to ovarian protection? While our data do not establish direct causality, we propose several potential mechanisms via the “gut-ovary axis” that are supported by our PICRUSt2-based functional prediction and correlation network analysis: (1) Reduced systemic inflammation: A healthy gut microbiota maintains intestinal barrier integrity, preventing translocation of lipopolysaccharide (LPS) and other bacterial products that trigger systemic low-grade inflammation and oxidative stress (Candelli et al., 2021). Our network analysis revealed significant positive correlations between DOP-enriched genera and pathways related to energy metabolism, which in turn correlated with favorable ovarian outcomes. (2) Production of bioactive metabolites: SCFAs produced by beneficial bacteria can enter the circulation and directly or indirectly (via the neuroendocrine system) modulate ovarian function, hormone secretion, and follicular development (Zhao et al., 2025; Morrison and Preston, 2016). Notably, DOP has been shown in other studies to enhance SCFA production through gut microbiota modulation (Xu et al., 2024; Tao et al.,2025). (3) Immune modulation: The gut microbiota shapes the systemic immune landscape, influencing ovarian immune cell populations and inflammatory cytokine profiles (Belkaid and Hand, 2014). While our study establishes a strong association between DOP-induced microbial shifts and ovarian protection, direct causal evidence requires further validation through fecal microbiota transplantation (FMT) or antibiotic-depletion experiments.

Integrated working model and clinical implications. Integrating these findings, we propose a working model: Radiation insult causes direct ovarian damage (ROS-mediated apoptosis, suppression of glycolysis-related markers, aberrant primordial follicle activation) and indirect damage via gut dysbiosis and systemic derangement. DOP intervenes through coordinated actions: (1) Direct ovarian effects: Scavenging ROS, inhibiting apoptosis, modulating PI3K/AKT/glycolysis-related markers in growing follicles, and suppressing pathological PI3K/AKT overactivation in primordial follicles. (2) Indirect systemic effects: Protecting the gut barrier, restoring microbial diversity and SCFA-producing bacteria, and mitigating systemic inflammation/oxidative stress. These interconnected pathways collectively support ovarian recovery and preservation of the follicle reserve.

### Limitations of the study

Limitations of the study. Several limitations should be acknowledged. First, although we demonstrated a strong association between gut microbiota restoration and ovarian protection, causality was not established. Fecal microbiota transplantation (FMT) or germ-free mouse models are needed to determine whether microbial shifts are a cause or a consequence of DOP’s protective effects. Second, the tested preparation had a polysaccharide content of 59.09%, meaning that a considerable proportion may consist of other unidentified components. While our characterization is consistent with previously reported DOP preparations (Tang et al., 2017; Feng et al., 2022; Hu et al., 2024), additional structural characterization (FTIR, NMR) and purity assessment (protein, nucleic acid, and endotoxin testing) would strengthen the reproducibility and translational potential of our findings. Throughout this manuscript, we have described the tested material as a partially purified polysaccharide-rich preparation and have avoided attributing all biological effects exclusively to polysaccharides, as the contribution of non-polysaccharide components cannot be excluded. Third, a DOP-only control group (non-irradiated mice receiving DOP) was not included in the *in vivo* study. Such a control would have been valuable to determine whether DOP itself affects ovarian function, hormone levels, follicle activation, glycolysis-related markers, or gut microbiota in the absence of irradiation. We acknowledge this limitation and plan to include this control in future studies. Fourth, the optimal dosing regimen, treatment duration, and potential toxicity of DOP require further evaluation in preclinical studies. Fifth, the specific polysaccharide fraction(s) responsible for the observed bioactivities were not identified; DOP is a complex mixture, and different fractions may exert distinct effects. Sixth, our *in vitro* experiments were limited to a single human granulosa cell line (KGN); validation in primary human granulosa cells or other ovarian cell types (e.g., oocytes, theca cells) would strengthen the findings. Seventh, the long-term effects of DOP on offspring health and fertility were not assessed, and while hormone recovery and follicle preservation suggest improvement in ovarian injury-related parameters, they do not establish preserved fertility. Functional reproductive outcomes such as mating success, pregnancy rate, litter size, oocyte quality, or embryo development would be required to definitively establish fertility preservation. Eighth, the whole-body irradiation model, while widely used, does not fully recapitulate the clinical scenario of localized pelvic radiotherapy; partial-body or ovarian-specific irradiation models should be considered in future studies. This limits the direct clinical translatability of our findings, as whole-body irradiation also causes systemic and gut injury, making it difficult to distinguish direct ovarian protection from broader systemic radioprotection. Ninth, the sample size for gut microbiota analysis was relatively small, and larger cohorts with longitudinal sampling would provide more robust conclusions. Tenth, the pretreatment (prophylactic) design mainly evaluates prevention rather than treatment of established radiation-induced POI; therapeutic-only (post-irradiation) administration would be more clinically relevant for patients who have already received radiotherapy. Eleventh, the Western blot data presented in this manuscript used trimmed blots; we have requested a 1-month extension to provide full uncropped Western blot images and molecular-weight markers in the supplementary material. Twelfth, the transient reduction in food intake during the early post-irradiation period could have influenced metabolism or gut microbiota; although final body weight did not differ significantly among groups, we acknowledge this as a potential confounding factor.

## Conclusion

In conclusion, this study demonstrates that a partially purified DOP preparation is a potential natural agent against radiation-induced ovarian damage. Our findings suggest that DOP orchestrates a protective response through a multi-organ, multi-pathway network involving the direct amelioration of ovarian apoptosis, modulation of the PI3K/AKT pathway in a manner suggestive of stage-specific effects, upregulation of glycolysis-related marker expression, and remediation of gut microbiota dysbiosis. These findings indicate that DOP improves ovarian endocrine, histological, and molecular parameters associated with radiation-induced ovarian injury. Future research should focus on identifying the active polysaccharide fractions, validating the causal role of specific microbial shifts through FMT experiments, including appropriate control groups, assessing functional reproductive outcomes, and exploring its efficacy in combination with established radioprotectants in clinically relevant irradiation models.

## Data Availability

The original contributions presented in this study are included in the article and its supplementary materials. The 16S rRNA sequencing data generated in this study have been deposited in the Genome Sequence Archive (GSA), National Genomics Data Center (NGDC) under accession number CRA045419 (available at: https://ngdc.cncb.ac.cn/gsa/search?searchTerm=CRA045419). Further inquiries can be directed to the corresponding authors.

## Glossary

AKT: Protein kinase B
AMH: Anti-Müllerian hormone
ASV: Amplicon sequence variant
DAPI: 4′,6-Diamidino-2-phenylindole
DDX4: DEAD-box helicase 4
DOP: Dendrobium officinale polysaccharides
E2: Estradiol
FMT: Fecal microbiota transplantation
FOXO3a: Forkhead box O3a
FSH: Follicle-stimulating hormone
GLUT4: Glucose transporter type 4
GPC: Gel permeation chromatography
H&E: Hematoxylin and eosin
HK1: Hexokinase 1
HPLC: High-performance liquid chromatography
IF: Immunofluorescence
IHC: Immunohistochemistry
KGN: Human ovarian granulosa-like tumor cell line
LDHA: Lactate dehydrogenase A
LH: Luteinizing hormone
LPS: Lipopolysaccharide
MALLS: Multi-angle laser light scattering
NMDS: Non-metric multidimensional scaling
PCNA: Proliferating cell nuclear antigen
PCoA: Principal coordinates analysis
PI3K: Phosphoinositide 3-kinase
PKM2: Pyruvate kinase M2
PMP: 1-Phenyl-3-methyl-5-pyrazolone
POI: Premature ovarian insufficiency
qPCR: Quantitative real-time polymerase chain reaction
qRT-PCR: Quantitative real-time polymerase chain reaction
ROS: Reactive oxygen species
rps6: Ribosomal protein S6
SCFA: Short-chain fatty acid
TUNEL: Terminal deoxynucleotidyl transferase dUTP nick end labeling
