# Supplementary material for: Dendrobium officinale polysaccharides ameliorate radiation-induced premature ovarian insufficiency through coordinated modulation involving the gut microbiota-PI3K/AKT-glycolysis axis
Source: Front Pharmacol. 2026 Oct 14;17:1891795. doi: 10.3389/fphar.2026.1891795 (PMC13713469; doi:10.3389/fphar.2026.1891795)
Supplement: Supplementary file 1 [file Supplementaryfile1.docx]

**Figure s1. Original western blot for three repeats**

**Supplementary Figure S**. Protein band intensity was quantified by

densitometry, and normalized intensity ratios (fold change relative to the

control group) are shown in the corresponding main figures.
