# Supplementary material for: Dendrobium officinale polysaccharides ameliorate radiation-induced premature ovarian insufficiency through coordinated modulation involving the gut microbiota-PI3K/AKT-glycolysis axis
Source: Front Pharmacol. 2026 Oct 14;17:1891795. doi: 10.3389/fphar.2026.1891795 (PMC13713469; doi:10.3389/fphar.2026.1891795)
Supplement: Supplementary file 2 [file Table1.docx]

**Supplementary Table S1. qRT-PCR primers used in this study.**

| Gene | Forward primer (5′→3′) | Reverse primer (5′→3′) | Amplicon size (bp) | Accession no. |
| --- | --- | --- | --- | --- |
| PI3K | TGTGGCACAGACTTGGTGTT | TTCTTCCCTTGAGATGTCTCCC | 153 | NM_001024955.2 |
| AKT | ACTCATTCCAGACCCACGAC | CACAATCTCCGCACCATAGA | 131 | NM_001409449.1 |
| mTOR | ACACCCTCCATCCACCTCAT | TAGCGGATATCAGGGTCAGGA | 128 | NM_020009.2 |
| FOXO3a | GGATGCGTGGACCGACTT | GGTGGCTGGTCTGTTCTCC | 466 | NM_001376967.1 |
| Rps6 | AGAGTGGAAGGGTTATGTG | ATCTTGGGTGCTTTGGT | 399 | NM_009096.3 |
| GLUT4 | CCAACTGGACCTGTAACTTC | GTGAAGATGAAGAAGCCAAG | 115 | XM_006532655.4 |
| HK1 | ACATCCTGATTGACTTCACC | TAATCGGTCACTCTCGATCT | 119 | XM_006513247.2 |
| PKM2 | TAGTTCTCACGGAGTCTGGC | AGCACGGCATCCTTACAC | 151 | NM_001405491.1 |
| LDHA | ACTGTGTAACTGCGAACTCC | GGGAATGATGAACTTGAAGA | 119 | NM_001136069.2 |
| GAPDH | ATTGTCAGCAATGCATCCTG | ATGGACTGTGGTCATGAGCC | 102 | NM_001411843.1 |

**Note:** All primers are shown 5′→3′. GAPDH was used as the internal reference gene. These primers were used for qRT-PCR in mouse ovarian tissues.
